# Induction of reactive oxygen intermediates in human monocytes by tumour cells and their role in spontaneous monocyte cytotoxicity

**DOI:** 10.1038/sj.bjc.6690118

**Published:** 1999-02

**Authors:** B Mytar, M Siedlar, M Woloszyn, I Ruggiero, J Pryjma, M Zembala

**Affiliations:** Department of Clinical Immunology, Polish–American Institute of Paediatrics, Jagiellonian University Medical College, Wielicka 265, Cracow, 30-663, Poland; Department of Microbiology and Immunology, Institute of Molecular Biology, Jagiellonian University, Cracow, Poland

**Keywords:** monocyte, tumour cells, interactions, reactive oxygen intermediates, cytotoxicity

## Abstract

The present study examined the ability of human monocytes to produce reactive oxygen intermediates after a contact with tumour cells. Monocytes generated oxygen radicals, as measured by luminol-enhanced chemiluminescence and superoxide anion production, after stimulation with the tumour, but not with untransformed, cells. The use of specific oxygen radical scavengers and inhibitors, superoxide dismutase, catalase, dimethyl sulphoxide and deferoxamine as well as the myeloperoxidase inhibitor 4-aminobenzoic acid hydrazide, indicated that chemiluminescence was dependent on the production of superoxide anion and hydroxyl radical and the presence of myeloperoxidase. The tumour cell-induced chemiluminescent response of monocytes showed different kinetics from that seen after activation of monocytes with phorbol ester. These results indicate that human monocytes can be directly stimulated by tumour cells for reactive oxygen intermediate production. Spontaneous monocyte-mediated cytotoxicity towards cancer cells was inhibited by superoxide dismutase, catalase, deferoxamine and hydrazide, implicating the role of superoxide anion, hydrogen peroxide, hydroxyl radical and hypohalite. We wish to suggest that so-called ‘spontaneous’ tumoricidal capacity of freshly isolated human monocytes may in fact be an inducible event associated with generation of reactive oxygen intermediates and perhaps other toxic mediators, resulting from a contact of monocytes with tumour cells. © 1999 Cancer Research Campaign


*British Journal of Cancer* (1999) **79**(5/6), 737–743
© 1999 Cancer Research Campaign
Article no. bjoc.1998.0118

### Induction of reactive oxygen intermediates in human monocytes by tumour cells and their role in spontaneous monocyte cytotoxicity

**B Mytar[1], M Siedlar[1], M Wołoszyn[1], I Ruggiero[1], J Pryjma[2] and M Zembala[1]**

[1]Department of Clinical Immunology, Polish–American Institute of Paediatrics, Jagiellonian University Medical College, Wielicka 265, 30-663 Cracow, Poland; and [2]Department of Microbiology and Immunology, Institute of Molecular Biology, Jagiellonian University, Cracow, Poland

**Summary** The present study examined the ability of human monocytes to produce reactive oxygen intermediates after a contact with tumour cells. Monocytes generated oxygen radicals, as measured by luminol-enhanced chemiluminescence and superoxide anion production, after stimulation with the tumour, but not with untransformed, cells. The use of specific oxygen radical scavengers and inhibitors, superoxide dismutase, catalase, dimethyl sulphoxide and deferoxamine as well as the myeloperoxidase inhibitor 4-aminobenzoic acid hydrazide, indicated that chemiluminescence was dependent on the production of superoxide anion and hydroxyl radical and the presence of myeloperoxidase. The tumour cell-induced chemiluminescent response of monocytes showed different kinetics from that seen after activation of monocytes with phorbol ester. These results indicate that human monocytes can be directly stimulated by tumour cells for reactive oxygen intermediate production. Spontaneous monocyte-mediated cytotoxicity towards cancer cells was inhibited by superoxide dismutase, catalase, deferoxamine and hydrazide, implicating the role of superoxide anion, hydrogen peroxide, hydroxyl radical and hypohalite. We wish to suggest that so-called ‘spontaneous’ tumoricidal capacity of freshly isolated human monocytes may in fact be an inducible event associated with generation of reactive oxygen intermediates and perhaps other toxic mediators, resulting from a contact of monocytes with tumour cells.

**Keywords:** monocyte; tumour cells; interactions; reactive oxygen intermediates; cytotoxicity

Macrophages are prominent in the cellular infiltrates surrounding the tumour and can constitute more than 50% of the total tumour mass (Leek et al, 1996). Mononuclear cell infiltration is regarded as a manifestation of the host response against the growing tumour (Zembala and Buckle, 1989). The anti-tumour effect of macrophages may be due to their cytotoxic/cytostatic activity, in which several toxic mediators including tumour necrosis factor α (TNF-α), reactive nitrogen intermediates (RNI) and reactive oxygen intermediates (ROI) are involved. The presence of TNF-mRNA has been demonstrated in tumour infiltrating macrophages (TIM), which is taken as evidence for the local production of this and perhaps other cytokines with anti-tumour activities (Beissert et al, 1989). In humans, there is no evidence for the local production of oxygen radicals in the tumour bed, however, the role of ROI in the anti-tumour response in humans has been indirectly implicated by observations that myeloperoxidase-deficient individuals have an increased incidence of neoplasms (Lanza et al, 1987). The direct evidence for the importance of ROI in the killing of tumour cells comes from the cell-free systems generating hydrogen peroxide or superoxide anion ($O_2^{\cdot-}$) (Ioannidis and de Groot, 1993; Palomba et al, 1996). TIM from mouse mammary carcinomas are able to produce ROI (Mantovani et al, 1992). An increased production of ROI by monocytes was observed in patients with cancer (Trulson et al, 1988, 1994), but the stimulus responsible for their generation has not been established. In contrast to murine macrophages, freshly isolated human monocytes exhibit considerable spontaneous cytotoxicity towards tumour cells (Davies and Edwards, 1992; Martin and Edwards, 1993), but its origins remain largely unclear. Davies and Edwards (1992) demonstrated that cytotoxic factors were released by monocytes cultured with K562 tumour cells, whereas monocytes and tumour cells cultured alone released factor(s) which promoted K562 cells growth. Furthermore, inhibitors of ROI added to K562 cells enhanced their growth. This led us to explore the possibility that spontaneous cytotoxic activity of monocytes may, at least partly, be due to ROI production induced by tumour cells in the co-culture.

The present study shows that stimulation of monocytes with tumour, but not untransformed, cells induces the production of ROI, as determined by the generation of chemiluminescence (CL) and $O_2^{\cdot-}$. The CL response was associated with $O_2^{\cdot-}$ and $OH^{\cdot}$ production and was dependent on the presence of myeloperoxidase. This study also demonstrates that $O_2^{\cdot-}$, hydrogen peroxide, $OH^{\cdot}$ and probably hypohalite(s) are involved in monocyte-mediated spontaneous cytotoxicity. This may imply that this ‘spontaneous’ cytotoxicity is probably an inducible event that occurs after contact of monocytes with tumour cells.

*Received 14 April 1998*
*Revised 13 July 1998*
*Accepted 16 July 1998*

*Correspondence to:* M Zembala, Department of Clinical Immunology, Polish–American Institute of Paediatrics, Jagiellonian University Medical College, Wielicka 265, 30-663 Cracow, Poland

### MATERIALS AND METHODS

### Isolation of cell populations

Human peripheral blood mononuclear cells were isolated from EDTA–blood of healthy donors on a standard Ficoll/Isopaque

(Pharmacia, Uppsala, Sweden) density gradient centrifugation. Monocytes were separated from mononuclear cells by counterflow centrifugal elutriation with a JE –5.0 elutriation system equipped with a 5-ml Sanderson separation chamber (Beckman, Palo Alto, CA, USA) as previously described (Zembala et al, 1994*a*). Isolated monocytes were 90–96% pure, as judged by flow cytometry analysis (FACScanV, Becton-Dickinson, Mountain View, CA, USA), using anti-CD14 (Leu-M3; Becton-Dickinson) monoclonal antibody (Zembala et al, 1994*a*) and contained 2–4% NK cells, as judged by staining with anti-CD56 monoclonal antibody (Dako, Glostrup, Denmark). The cells were suspended in culture medium RPMI-1640 (Biochrom, Berlin, Germany) with gentamycin (25 μg $ml^{-1}$, Biochrom), glutamine (2 mM, Gibco, Paisley, UK) and 10% fetal calf serum (FCS) (Biochrom).

### Cell lines

The following human cell lines were used: HPC-4 (human pancreatic adenocarcinoma) (Siedlar et al, 1995), CaOV (ovarian carcinoma), normal human skin fibroblasts, peritoneal mesothelial cells (obtained in our laboratory), and established cell lines DeTa (colon carcinoma), Hu 1703 (transitional cell bladder carcinoma), BC3726 (v-raf transfected normal bladder epithelium) and HCV 29 (normal uroepithelium) (Zembala et al, 1994*a*). Hep G2 (hepatocellular carcinoma) and Caco-2 (colon adenocarcinoma) were obtained from Professor V Colizzi (II Universitá di Roma, Italy). Cell lines were cultured by biweekly passages in RPMI-1640 with 5% FCS. All cell lines were regularly tested for *Mycoplasma* sp. contamination using standard PPLO medium (Difco, Detroit, MI, USA).

### Chemiluminescence

CL was used to detect activated oxygen species (Ernst et al, 1984). Various tumour cells/monocytes ratios were used to establish conditions for optimal CL response. As a result, $1 \times 10^5$ monocytes and $5 \times 10^5$ tumour cells were resuspended in 100 μl of culture medium and added to the vials (Berthold, Vienna, Austria). Then, 300 μl of 2 mM luminol (5-amino-2,3-dihydro-1,4-phtalazinedione, Sigma, St. Louis, MO, USA) in Krebs-Ringer buffer with $Mg^{2+}$ and $Ca^{2+}$ was added. The vials were placed in the measuring chambers of a Multi-Biolumat (Berthold), kept at 37°C and the CL response was continuously recorded. The results were calculated as integrals (cumulative counts, cc) of the response recorded during 300 min. Data were expressed as the percentage of control response or CL index, calculated as:

$$\text{CL index} = \frac{\text{cumulative counts of tumour-stimulated monocytes}}{\text{cumulative counts of unstimulated monocytes}}$$

In some experiments, phorbol myristate acetate (PMA) (100 ng $ml^{-1}$, Sigma) was added to monocytes or tumour cell suspension and the CL response was recorded.

### Measurement of $O_2^-$ production

Superoxide production was determined by the cytochrome *c* reduction assay as described by Pick and Mizel (1981). Briefly, monocytes ($1 \times 10^5$ per well) and tumour cells ($1 \times 10^4$–$3 \times 10^5$ per well) were resuspended in 160 μM solution of ferricytochrome *c* (Sigma) in phenol-red-free Hanks' balanced salt solution (HBSS), and plated in 96-well flat bottom tissue culture plates (Nunc, Roskilde, Denmark) in a final volume of 100 μl per well. Wells supplemented with 300 U $ml^{-1}$ of superoxide dismutase (SOD) (Sigma) were used as blanks. Cytochrome *c* reduction was measured after 2 h of incubation at 550 nm in an Elisa reader (Labsystems Multiscan Plus, Labsystems, Finland) using a 450-nm reference filter (Martin and Edwards, 1993). Results were expressed as nM per $1 \times 10^5$ cells per 2 h.

### Determination of hydrogen peroxide production

Hydrogen peroxide detection was based on the horseradish peroxidase-dependent oxidation of phenol red, as described by Pick and Mizel (1981). Briefly, cells were washed and resuspended in assay solution containing 0.56 mM of phenol red (Sigma) and 20 U $ml^{-1}$ horseradish peroxidase (Sigma) in phenol-red-free HBSS, and seeded in tissue culture plates in a final volume of 100 μl per well. After incubation for 2 h, the reaction was stopped by the addition of 10 μl of 1 N sodium hydroxide per well and absorbance was read at 600 nm. Wells with sodium hydroxide added at the beginning of testing were used as blanks. Results were expressed as nM per $1 \times 10^5$ cells per 2 h.

### Flow cytometry and cell sorting

Monocytes ($2 \times 10^6$) were stimulated with HPC-4 cells ($5 \times 10^6$) for 40 min at 37°C. After washing, the cells were stained with anti-CD14 monoclonal antibody (Becton-Dickinson) for 20 min at 4°C. Then, cells were washed and resuspended in 5 ml of phosphate-buffered saline (PBS). Cell sorting was performed with FACS Vantage Cytometer (Becton-Dickinson) equipped with a Power Macintosh 7600/120 computer using a Cell Quest v. 3.0 software. The ion laser Innova Enterprice II (Coherent, Santa Clara, CA, USA) operating at 488 nm was used as a light source. After setting $CD14^+$ (monocytes) and $CD14^-$ (tumour cells) gating, sorting was performed using a 70 μm nozzle tip with a drop drive frequency of 25 kHz, three drops envelopes and 'normal-R' sort mode. Sorted cells were collected into water-cooled (constant temperature circulator, Neslab Instruments, Portsmouth, NH, USA) polystyrene Falcon 2057 tubes (Becton-Dickinson), precoated with FCS to avoid plastic charging and cell attachment to the well. The purity of sorted cells was checked by flow cytometry and it exceeded 95%.

### Inhibitors of ROI production

The following blockers and scavengers of oxygen-free radicals (all from Sigma) were used at the indicated concentrations: superoxide dismutase (SOD, 100–600 U $ml^{-1}$), catalase (CAT, 100–5000 U $ml^{-1}$), dimethyl sulphoxide (DMSO, 50–200 mM), deferoxamine mesylate (25–200 mM) and the inhibitor of myeloperoxidase 4-aminobenzoic acid hydrazide (ABAH, 50–150 μM).

### Cytotoxicity assay

To obtain a significant cytotoxicity, monocytes were cultured with tumour cells at 1:0.4 ratio (Davies and Edwards, 1992). Monocytes ($5 \times 10^4$ per well), HPC-4 ($2 \times 10^4$ per well) or their mixtures were cultured for 20 h in the presence or absence of ROI inhibitors or scavengers. Then, the culture medium was removed

*© Cancer Research Campaign 1999*

and 100 μl per well of MTT (2 mg ml$^{-1}$, 3-[4,5-dimetylthiazol-2-yl]-2,5-diphenyltetrazolium bromide, Sigma) dye solution was added. Cell proliferation was assessed by reduction of MTT, as previously described (Zembala et al, 1993; see also Hruby and Beck, 1997). Percentage of cytotoxicity was calculated according to the formula previously described (Hruby and Beck, 1997):

$$\left(1 - \frac{\text{OD (monocytes + tumour cells)} - \text{OD (monocytes alone)}}{\text{OD (tumour cells alone)}}\right) \times 100$$

The effect of inhibitors on tumour cell survival was calculated as:

$$\frac{\text{OD (tumour cells + inhibitor)}}{\text{OD (tumour cells alone)}} \times 100$$

### Statistical analysis

Significance analysis was performed using an unpaired Student's *t*-test. *P*-values < 0.05 were considered significant.

### RESULTS

### Induction of chemiluminescent response of human monocytes by tumour cells

Monocytes were incubated with various tumour cells in the presence of luminol, and the CL was recorded in a chemiluminometer. Figure 1A shows that monocytes incubated with cancer cells (HPC-4 or DeTa) gave rise to the CL response, which reached a peak around 100–120 min, whereas monocytes kept in the medium yielded negligible response. The addition of normal human uroepithelial cells induced low level of CL. The response to tumour cells was much slower than that seen after stimulation of monocytes with PMA (Figure 1B), which peaked at 3–8 min, whereas during the first 30 min monocytes stimulated with HPC-4 cells generated little CL. Also, no significant CL was recorded when unstimulated and PMA-stimulated tumour cells alone were used. Tumour cell lines differed in their ability to evoke CL response. As shown in Table 1, a strong response was induced by HPC-4, DeTa and Hep G2 cell lines. Other cancer cells, Caco-2 and Hu 1703, elicited a moderate CL response, whereas BC 3726 and CaOV induced a weak response. In contrast, normal uroepithelial cells, peritoneal mesothelial cells or fibroblasts (Table 1) did not stimulate significant CL. It was concluded that the CL response of monocytes was the result of their direct interaction with tumour cells, and that the response was different, but characteristic, for the given cell line used.

### Identification of cells responsible for CL generation

Because, potentially, both monocytes and cancer cells can produce ROI, attempts were made to establish which cell type was responsible for the CL response. Monocytes were stimulated with tumour cells for 40 min, stained with anti-CD14 monoclonal antibody and sorted out into CD14$^+$ and CD14$^-$ populations. As control, unstimulated monocytes were also sorted ('dummy sorting'). These procedures were timed in such a way to measure CL around the peak of response. Table 2 shows that CD14$^+$ cells that were in contact with tumour cells gave a significantly higher CL response than 'dummy sorted' CD14$^+$ cells. CD14$^-$ cells showed a background CL. This clearly indicates that monocytes and not tumour cells are the source of luminol-dependent CL.

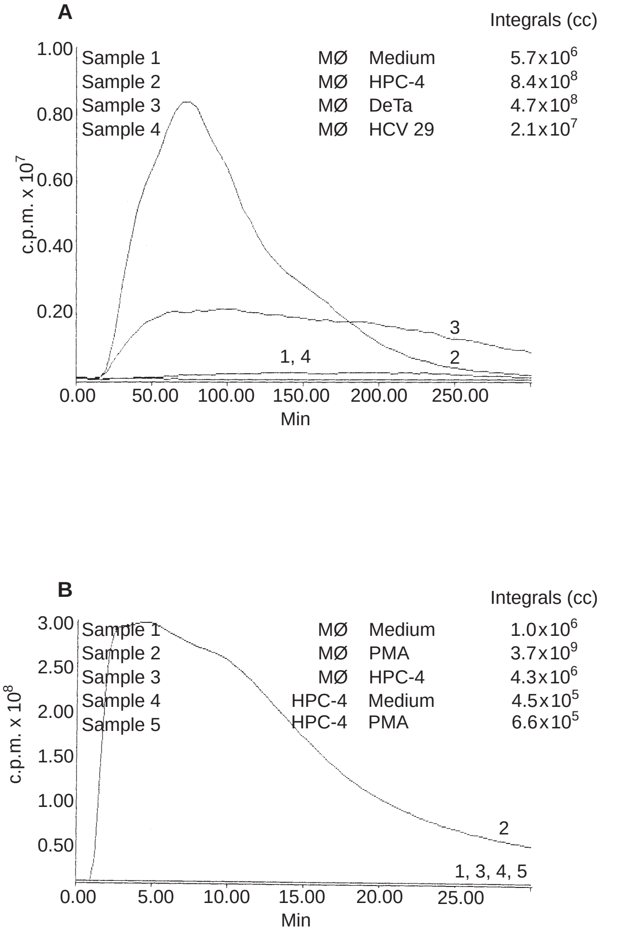

**Figure 1** The kinetics of the CL response of monocytes stimulated with tumour cells. (**A**) Monocytes (1 × 10$^5$) were mixed with tumour cells (5 × 10$^5$) HPC-4 (pancreatic carcinoma), DeTa (colon carcinoma) and normal uroepithelial cells (HCV 29). After addition of luminol, CL was recorded over 300 min. (**B**) Monocytes were stimulated with PMA (1 hg ml$^{-1}$) or HPC-4 cells and CL recorded during 30 min. Unstimulated or PMA-stimulated HPC-4 cells were also used. Results calculated as integrals and expressed in cc (cumulative counts) are also indicated. A typical experiment, one of four, is shown

**Table 1** Ohemiluminescent response of monocytes stimulated with different cell lines

| Cell line | CL index (mean ± s.d.) | Range | Number of experiments |
|---|---|---|---|
| HPC-4 | 26.5 ± 17.5 | 9.0–58.0 | 10 |
| Hep G2 | 25.3 ± 6.7 | 20.7–33.0 | 4 |
| DeTa | 14.1 ± 9.7 | 4.7–32.5 | 7 |
| Caco-2 | 7.2 ± 5.7 | 2.0–5.3 | 4 |
| Hu 1703 | 6.4 ± 3.5 | 3.8–10.4 | 3 |
| BC 3726 | 4.2 ± 3.1 | 1.3–7.6 | 3 |
| CaOV | 4.1 ± 0.4 | 3.7–4.5 | 2 |
| HCV 29 | 2.7 ± 1.0 | 1.4–4.1 | 5 |
| Mesothelial cells | 2.8 ± 0.2 | 2.8–3.1 | 2 |
| Skin fibroblasts | 2.6 ± 0.6 | 2.0–3.3 | 4 |

Monocytes (1 × 10$^5$ cells in 50 μl of culture medium) were stimulated with tumour cells (5 × 10$^5$ cells in 50 μl of culture medium) in Krebs-Ringer buffer with 2 mM luminol, and chemiluminescence (CL) was recorded during 300 min. The data were expressed as index of CL, as described in Materials and methods.

© Cancer Research Campaign 1999 

**Table 2** Chemiluminescence of monocytes (MO) sorted after contact with tumour cells

| Cells | | Integrals (cc) |
|---|---|---|
| MO, medium/unsorted | | $0.90 \times 10^7$ |
| MO, medium/sorted $CD14^+$ | | $0.75 \times 10^7$ |
| MO, tumour cells/sorted | $CD14^+$ | $7.49 \times 10^7$ |
| | $CD14^-$ | $0.08 \times 10^7$ |

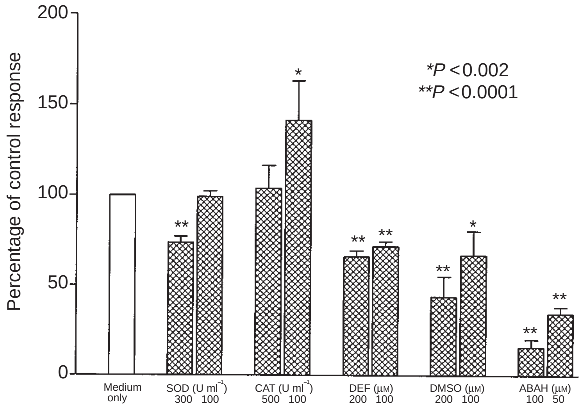

**Figure 2** Effect of ROI inhibitors on CL response of monocytes stimulated with HPC-4 cells. SOD, catalase (CAT), deferoxamine (DEF), dimethyl sulphoxide (DMSO) and ABAH were added to the mixture of monocytes and HPC-4 cells. The results are expressed as the percentage of control response of monocytes and HPC-4 cells without inhibitors. Means ± s.e. of six different experiments are shown

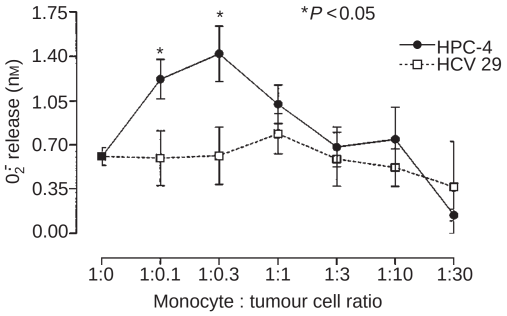

**Figure 3** Generation of $O_2^{\cdot -}$ release by monocytes ($1 \times 10^5$ per well) stimulated with different numbers of cancer (HPC-4) or normal uroepithelial (HCV 29) cells. The level of $O_2^{\cdot -}$ was determined after 2 h of incubation. Data, based on four experiments, are expressed as nM per 2 h (means ± s.e.)

### The type(s) of free radical(s) generated by monocytes stimulated with tumour cells

To establish which type of ROI was associated with generation of CL by monocytes, the blockers or scavengers of free oxygen radicals were used. SOD, which dismutates $O_2^{\cdot -}$ to hydrogen peroxide, deferoxamine, which prevents $\dot{H}O$ formation, and DMSO, scavenger of $OH^{\cdot}$, diminished CL response of monocytes stimulated with HPC-4 cells (Figure 2). This indicates that mainly $O_2^{\cdot -}$ and $OH^{\cdot}$ were detected by luminol-dependent CL. Surprisingly, catalase, which decomposes hydrogen peroxide to water and oxygen, had no effect on CL generation, indicating that hydrogen peroxide was not a major type of ROI evoking CL. Also, no synergistic effect of SOD and catalase was observed (data not shown). The CL response was myeloperoxidase-dependent because it was markedly inhibited by ABAH. This indirectly indicated that luminol-enhanced CL of monocytes stimulated with tumour cells is dependent on $O_2^{\cdot -}$ and $OH^{\cdot}$ production and the presence of myeloperoxidase.

### Demonstration of $O_2^{\cdot -}$, but not hydrogen peroxide, production by monocytes stimulated with tumour cells

The cytochrome *c* reduction test was used to measure the level of $O_2^{\cdot -}$ production in the culture of monocytes with tumour cells within 30–180 min. Addition of tumour cells at a low ratio (1:0.1–1.0; monocytes:tumour cells respectively) increased $O_2^{\cdot -}$ production above the background level (release of $O_2^{\cdot -}$ by unstimulated monocytes) (Figure 3), whereas the admixure of higher numbers of HPC-4 resulted in a decreased response. HCV 29 cells, incapable of stimulating CL in monocytes (Figure 1, Table 1), were used as control.

When monocytes were cultured with tumour cells for 40 min and then sorted into $CD14^+$ and $CD14^-$ populations, it was observed that the highest amount of $O_2^{\cdot -}$ was produced by $CD14^+$ cells (3.3 nM ± 0.08 per $1 \times 10^5$ cells, mean of three different experiments), whereas $CD14^-$ cells generated 1.06 nM and 'dummy sorted' $CD14^+$ cells – 0.90 nM of $O_2^{\cdot -}$. In these experiments, the mean level of $O_2^{\cdot -}$ found in the co-culture was 1.44 nM. The production of $O_2^{\cdot -}$ by $CD14^-$ cells was probably connected with the presence of small numbers of contaminating monocytes in this population because HPC-4 cells alone, unstimulated or stimulated with PMA (Figure 1B), LPS or fMLP (data not shown) did not produce $O_2^{\cdot -}$. This again clearly points to $CD14^+$ cells as the source of $O_2^{\cdot -}$ in the co-culture. In contrast, hydrogen peroxide was spontaneously produced by both monocytes and, even more, by tumour cells. The level of hydrogen peroxide was not increased in co-cultures (data not shown), in keeping with the absence of catalase effect on the generation of CL by monocytes.

### The effect of ROI scavengers on the inhibition of tumour cell growth by monocytes

Freshly isolated human monocytes exhibit considerable cytotoxicity towards tumour cells. To assess a possible role of ROI in this phenomenon, several ROI inhibitors were added to monocytes cultured with HPC-4 tumour cells at the ratio 1:0.4. Under these conditions, cytotoxicity, as assessed by the MTT test, in untreated cultures was approximately 33%. It was significantly lower when cells were treated with SOD, catalase and deferoxamine (Figure 4A). Also, ABAH diminished cytotoxicity. These inhibitors had no effect on the proliferation of tumour cells cultured alone (Figure 4B). This implies that hydrogen peroxide, $O_2^{\cdot -}$ and $OH^{\cdot}$ were involved in monocyte-mediated cytocidal activity.

### DISCUSSION

The generation of ROI is thought to be one of the mechanisms responsible for the anti-tumour effect of activated macrophages (Ding et al, 1988; Jaattela and Wissing, 1993). It is also proposed that the cytotoxic effect of TNF produced by monocytes is due to the generation of ROI within target cells, which in turn activate

 © Cancer Research Campaign 1999

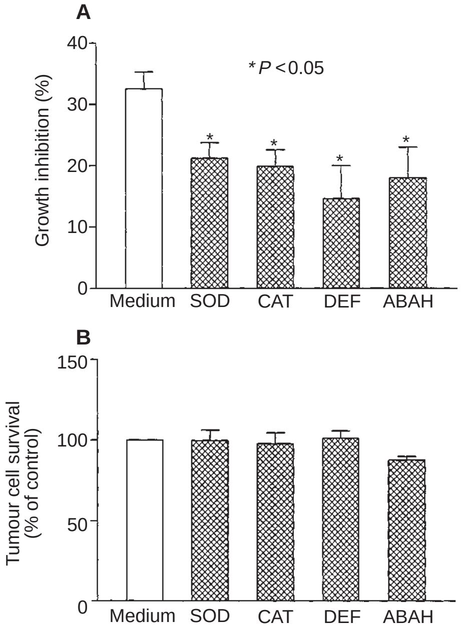

**Figure 4** Effect of ROI inhibitors on the growth of HPC-4 cells cultured with or without monocytes. SOD (300 U $ml^{-1}$), catalase (CAT, 500 U $ml^{-1}$), deferoxamine (DEF, 100 mM) and ABAH (100 mM) were added to co-cultures of monocytes ($5 \times 10^4$ per well) and HPC-4 cells ($2 \times 10^4$ per well) (**A**), or to HPC-4 cells cultured alone (**B**). After 24 h of incubation, MTT was added for a further 2 h as described in Materials and methods. The results are expressed as the percentage of growth inhibition of tumour cells by monocytes (**A**) or tumour cell survival (**B**). The data represent the mean values of three independent experiments ± s.e.

pre-existing toxic mediators, e.g. proteases (Wong and Goeddel, 1989). There is also direct evidence that generation of $O_2^{\bullet}$ and hydrogen peroxide in cell-free systems causes tumour cell killing (Ioannidis and de Groot, 1993; Palomba et al, 1996). In humans, there is indirect evidence that spontaneous cytotoxicity of monocytes towards tumour cells may be associated with their ability to produce ROI (Davies and Edwards, 1992; Martin and Edwards, 1993). Although an altered production of ROI by monocytes of cancer patients has been observed (Trulson et al, 1988, 1994; Hara et al, 1991), it is unclear which cells, and how, are stimulated for ROI production.

The present studies addressed the question of whether monocytes can be directly activated by tumour cells for ROI generation. We are aware of only one report on the ability of K562 (chronic myelogenous leukaemia) cells to stimulate peripheral blood mononuclear cells for a CL response (Ernst et al, 1984). In this report, monocytes were indirectly implicated as a source of CL, because phagocyte-depleted mononuclear cells were poor producers. Our study clearly demonstrates that monocytes and not tumour cells are the source of CL because $CD14^+$, but not $CD14^-$, cells sorted out from the co-culture produced CL and released $O_2^{\bullet}$. The role of natural killer (NK) cells in the tumour cell-induced CL response is unlikely because contamination of elutriated monocytes with $CD56^+$ cells is low, approximately only 2% $CD14^+$ cells show expression of CD56 (Rothe et al, 1996), and, finally, cell populations highly enriched for NK activity do not respond in target cell- and latex-induced CL (Ernst et al, 1984). Tumour cell-induced CL response peaked around 2 h. These kinetics are distinct from the rapid CL response seen after the activation of monocytes with PMA (Figure 1B), lipoplysaccharide (LPS), latex or zymosan, which lasts for 1–15 min (Trulson et al, 1988; Martin and Edwards, 1993). Monocytes responded poorly to normal cells, such as skin fibroblasts, uroepithelial and mesothelial cells, compared with cancer cells. This may suggest that monocytes are able to discriminate between transformed and normal cells. This is in keeping with previous findings regarding the ability of monocytes to release TNF and nitric oxide after stimulation with cancer, but not untransformed, cells (Zembala et al, 1994*a*, *b*; Hasday et al, 1990), and other observations on the capacity of activated monocytes to lyse tumour, but not non-neoplastic, cells (Galligioni et al, 1993). The molecular mechanism(s) by which monocytes can distinguish cancer cells remains largely unknown, but the role of phosphatidylserine (Utsugi et al, 1991) or hyaluronic acid (Pericle et al, 1996) on tumour cells and CD44 determinants on monocytes (Zembala et al, 1994*b*) have been implicated.

Why various tumour cells possess different capacities to induce the CL response is unclear. Distinct surface structures of tumour cells were found to be responsible for monocyte activation for TNF production (Jänicke and Männel, 1990), but their molecular structure has not been identified. Our preliminary observations indicate that the presence of some adhesion molecules, including CD44, and expression of hyaluronic acid on tumour cells, which is known to vary considerably (van Muijen et al, 1995), may be important.

CL is commonly used for the detection of ROI production. However, there is no agreement on the type(s) of the oxygen species responsible for generation of CL. Hydrogen peroxide, $O_2^{\bullet}$, ${}^1O_2$ (singlet oxygen) or $OH^{\bullet}$ is generated during oxidative burst. The decrease of tumour cell-induced CL after treatment with deferoxamine or DMSO points to the role of $OH^{\bullet}$. In the present system, CL was dependent on the presence of myeloperoxidase, as shown by the inhibitory effect of ABAH. The inhibition of CL by SOD indicated that also $O_2^{\bullet}$ is involved. Hydrogen peroxide, $O_2^{\bullet}$, and myeloperoxidase are implicated in CL generated by monocytes stimulated with PMA and fMLP (McNally and Bell, 1996). However, in our hands, CL induced by tumour cells was not dependent on hydrogen peroxide generation because catalase had no effect. Hence, tumour cell-induced CL response of monocytes may be distinct from that induced by PMA or fMLP because of different kinetics and range of oxygen species generated.

The involvement of $O_2^{\bullet}$ in the CL response was proven by a direct demonstration of $O_2^{\bullet}$ production by monocytes stimulated with cancer but not normal epithelial cells. Surprisingly, this production occurred after stimulation of monocytes with rather low numbers of tumour cells. The increased proportion of the latter resulted in a smaller $O_2^{\bullet}$ release, which can be due to a negative (inhibitory or inactivating) influence of tumour cells. It is known that cancer cells may produce several factors that inactivate toxic mediators, including SOD (Sun et al, 1989). Transforming growth factor β (TGF-β), produced by tumour cells, may also be responsible for the inhibition of $O_2^{\bullet}$ release because it blocks signalling pathways, including protein tyrosine kinase (Pazdrak et al, 1995) which is involved in signal transduction for ROI generation (Mytar et al, 1998). Studies are underway to define inhibitory factors of tumour cells which may down-regulate the response of monocytes.

In contrast to murine macrophages, freshly isolated human blood monocytes exhibit considerable spontaneous cytotoxicity towards tumour cells (Davies and Edwards, 1992), and there is indirect evidence suggesting that ROI may be involved (Martin

*© Cancer Research Campaign 1999*

and Edwards, 1993). On the basis of our study, it appears that this ‘spontaneous’ cytotoxicity of monocytes may in fact be due to induction of production of several toxic molecules after contact with tumour cells. As a result, generation of TNF (Hasday et al, 1990; Zembala et al, 1994*b*), RNI (Zembala et al, 1994*a*) and ROI (this study) may be initiated and be responsible for the inhibition of proliferation and/or destruction of tumour cells by previously non-activated monocytes. The present study indicates that $O_2^{\cdot}$, hydrogen peroxide and $OH^{\cdot}$ are involved in monocyte-mediated cytotoxicity towards tumour cells. This conclusion is based on the effect of specific ROI inhibitors. Because these inhibitors had no effect on proliferation of tumour cells in the absence of monocytes, it is likely that they down-regulated ROI production by monocytes. The effect of catalase, the specific scavenger of hydrogen peroxide, is somewhat confusing. Because highly toxic hypohalites and $OH^{\cdot}$ are generated from hydrogen peroxide (King et al, 1997), the question arises whether inhibitory action of catalase on the cytocidal activity of monocytes may be due to decreased generation of these toxic molecules. The role of hypohalites may be supported by the inhibitory effect of ABAH, which by blocking myeloperoxidase activity prevents their generation. It remains unclear whether all above radicals are effector molecules or whether at least some of them ($O_2^{\cdot}$, hydrogen peroxide) act mainly as precursors of other toxic intermediates.

The ability of various tumour cell lines to evoke a CL response of monocytes may perhaps explain why in different systems cytotoxicity of monocytes was found to be ROI-dependent or ROI-independent (Nakabo and Pabst, 1997). The involvement of ROI in the cytotoxic effect of Bacille Calmette-Guérin (BCG)- or PMA-activated murine macrophages (Nathan et al, 1979; Nathan and Cohn, 1980) and LPS- or PMA-activated human monocytes (Klassen and Sagone, 1980; Mavier and Edgington, 1984; Martin and Edwards, 1993; McLachlan et al, 1995) is well established. The present data suggest that contact with tumour cells may activate cytocidal capacity of monocytes and generation of ROI. Hence, ‘spontaneous’ monocyte cytotoxicity may, in fact, be an inducible event. The question arises whether TIM can also be stimulated by cancer cells for generation of ROI in the tumour bed.

### ACKNOWLEDGEMENTS

This work was supported by the National Committee for Scientific Research (grant no. 4.05A. 084.12). We wish to thank Ms M Hyszko for skilful technical assistance and Ms L Czernicka for help in preparation of the manuscript.

### REFERENCES

Beissert S, Bergholz M, Waase I, Lepsien G, Schauer A, Pfizenmaier K and Ronke M (1989) Regulation of tumour necrosis factor gene expression in colorectal adenocarcinoma: in vivo analysis by in situ hybridization. *Proc Natl Acad Sci USA* **86**: 5064–5068

Davies B and Edwards SW (1992) Interactions between human monocytes and tumour cells. Monocytes can either enhance or inhibit the growth and survival of K562 cells. *Br J Cancer* **66**: 463–469

Ding AH, Nathan CF and Stuehr DJ (1988) Release of reactive nitrogen intermediates and reactive oxygen intermediates from mouse peritoneal macrophages. Comparison of activating cytokines and evidence for independent production. *J Immunol* **141**: 2407–2412

Ernst M, Lange A, Flad H-D, Havel A, Ennen J and Ulmer AJ (1984) Dissociation of responses measured by natural cytotoxicity and chemiluminescence. *Eur J Immunol* **14**: 634–639

Galligioni E, Quaia M, Spada A, Favaro D, Santarosa M, Talamini R and Monfardini S (1993) Activation of cytolytic activity in peripheral blood monocytes of renal cancer patients against non-cultured autologous tumour cells. *Int J Cancer* **55**: 380–385

Hara N, Ichinose Y, Asoh H, Yano T, Kawasaki M and Ohta M (1991) Superoxide anion – generating activity of polymorphonuclear leukocytes and monocytes in patients with lung cancer. *Cancer* **69**: 1682–1687

Hasday JD, Shah EM and Lieberman AP (1990) Macrophage tumour necrosis factor alfa release is induced by contact with some tumors. *J Immunol* **145**: 371–385

Hruby Z and Beck K-F (1997) Cytotoxic effect of autocrine and macrophage-derived nitric oxide on cultured rat mesangial cells. *Clin Exp Immunol* **107**: 76–82

Ioannidis M and de Groot H (1993) Cytotoxicity of nitric oxide in Fu5 rat hepatoma cells: evidence for co-operative action with hydrogen peroxide. *Biochem J* **296**: 341–345

Jaattela M and Wissing D (1993) Heat-shock proteins protect cells from monocyte cytotoxicity – possible mechanism of self protection. *J Exp Med* **177**: 231–236

Jänicke R and Männel D (1990) Distinct tumor cell membrane constituents activate human monocytes for tumor necrosis factor synthesis. *J Immunol* **144**: 1144–1150

King CC, Jefferson MM and Thomas EL (1997) Secretion and inactivation of myeloperoxidase by isolated neutrophils. *J Leukoc Biol* **61**: 293–302

Klassen DK and Sagone AL (1980) Evidence for both oxygen and non-oxygen dependent mechanisms of antibody sensitized target cell lysis by human monocytes. *Blood* **56**: 985–993

Lanza F, Fietta A, Spisani S, Castoldi GL and Traniello S (1987) Does a relationship exist between neutrophil myeloperoxidase deficiency and the occurrence of neoplasms? *J Clin Lab Immunol* **22**: 175–180

Leek RD, Lewis CE, Whitehouse R, Greenall M, Clarke J and Harris AL (1996) Association of macrophage infiltration with angiogenesis and prognosis in invasive breast carcinoma. *Cancer Res* **56**: 4625–4629

Mantovani A, Bottazzi B, Colotta F, Sozzani S and Ruco L (1992) The origin and function of tumour-associated macrophages. *Immunol Today* **13**: 265–270

Martin JHJ and Edwards SW (1993) Changes in mechanisms of monocyte/macrophage-mediated cytotoxicity during culture. Reactive oxygen intermediates are involved in monocyte-mediated cytotoxicity, whereas reactive nitrogen intermediates are employed by macrophages in tumour cell killing. *J Immunol* **150**: 3476–3486

Mavier P and Edington TS (1984) Human monocyte-mediated tumour cytotoxicity. I. Demonstration of an oxygen-dependent myeloperoxidase-independent mechanism. *J Immunol* **132**: 1980–1986

McLachlan JA, Serkin CD, Morrey KM and Bakouche O (1995) Antitumoral properties of aged human monocytes. *J Immunol* **154**: 832–834

McNally JA and Bell AL (1996) Myeloperoxidase-based chemiluminescence of polymorphonuclear leukocytes and monocytes. *J Biolumin Chemilumin* **11**: 99–106

Nakabo Y and Pabst MJ (1997) C2-ceramide and C6-ceramide inhibited priming for enhanced release of superoxide in monocytes, but had no effect on killing of leukaemic cells by monocytes. *Immunology* **154**: 477–482

Nathan CF and Cohn Z (1980) Role of oxygen-dependent mechanisms in antibody-induced lysis of tumour cells by activated macrophages. *J Exp Med* **152**: 198–206

Nathan CF, Silverstein SC, Brukner L and Cohn ZA (1979) Extracellular cytolysis by activated macrophages and granulocytes. II. Hydrogen peroxide as a mediator of cytotoxicity. *J Exp Med* **149**: 100–113

Palomba L, Sestili P, Cattabeni F, Azzi A and Cantoni O (1996) Prevention of necrosis and activation of apoptosis in oxidatively injured human myeloid leukemia U937 cells. *FEBS Lett* **390**: 91–94

Pazdrak K, Justement L and Alam R (1995) Mechanism of inhibition of eosinophil activation by transforming growth factor-b. *J Immunol* **155**: 4454–4458

Pericle F, Sconocchia G, Titus JA and Segal DM (1996) CD44 is a cytotoxic triggering molecule on human polymorphonuclear cells. *J Immunol* **157**: 4657–4663

Pick E and Mizel D (1981) Rapid microassays for the measurements of superoxide and hydrogen peroxide production by macrophages in culture using an automatic enzyme immunoassay reader. *J Immunol Methods* **46**: 211–226

Rothe G, Gabriel H, Kovacs E, Klucken J, Stöhr J, Kindermann W and Schmitz G (1996) Peripheral blood mononuclear phagocyte subpopulations as cellular markers in hypercholesterolemia. *Arteriosclerosis Thromb Vascular Biol* **16**: 1437–1447

Siedlar M, Stachura J, Szczepanik A, Mattei M, Popiela T, Vendetti S, Colizzi V and Zembala M (1995) Characterization of human pancreatic carcinoma cell line with high metastatic potential in SCID mice. *Invasion Metastasis* **15**: 60–69

 © Cancer Research Campaign 1999

Sun Y, Oberley LW, Elwell JH and Sierra-Rivera E (1989) Anti-oxidant enzyme activities in normal and transformed mouse liver cells. *Int J Cancer* **44**: 1028–1033

Trulson A, Nilsson S and Venge P (1988) Lucigenin-enhanced chemiluminescence in blood is increased in cancer. *Am J Clin Pathol* **91**: 441–445

Trulson A, Nilsson S, Brekkan E and Venge P (1994) Patients with renal cancer have a larger proportion of high-density blood monocytes with increased lucigenin-enhanced chemiluminescence. *Inflammation* **18**: 99–105

Utsugi T, Schroit AJ, Connor J, Bucana CD and Fidler IJ (1991) Elevated expression of phosphatidylserine in the outer membrane leaflet of human tumour cells and recognition by activated human blood monocytes. *Cancer Res* **51**: 3062–3066

Van Muijen GN, Danen EH, Veerkamp JH, Ruiter DJ, Lesley J and Van Den Heuvel LP (1995) Glycoconjugate profile and CD44 expression in human melanoma cell lines with different metastatic capacity. *Int J Cancer* **61**: 241–248

Wong GH and Goeddel D (1989) Tumour necrosis factor. In *Human Monocytes*. Zembala M and Asherson GL (eds), pp. 195–215, Academic Press: London

Zembala M and Buckle AM (1989) Monocytes in malignant disease. In *Human Monocytes*. Zembala M and Asherson GL (eds), pp. 513–528, Academic Press: London

Zembala M, Czupryna A, Wieckiewicz J, Jasiński M, Pryjma J, Ruggiero I, Siedlar M and Popiela T (1993) Tumour cell induced production of tumour necrosis factor by monocytes of gastric patients receiving BCG immunotherapy. *Cancer Immunol Immunother* **36**: 127–132

Zembala M, Siedlar M, Marcinkiewicz J and Pryjma J (1994*a*) Human monocytes are stimulated for nitric oxide release in vitro by some tumour cells but not by cytokines and lipopolysacharide. *Eur J Immunol* **24**: 435–439

Zembala M, Siedlar M, Ruggiero I, Wieckiewicz J, Mytar B, Mattei M, Colizzi V (1994*b*) The MHC class II and CD44 molecules are involved in the induction of tumour necrosis factor (TNF) gene expression by human monocytes stimulated with tumour cells. *Int J Cancer* **56**: 269–274

*© Cancer Research Campaign 1999*

## References

[PDF_00663] Beissert S., Bergholz M., Waase I., Lepsien G., Schauer A., Pfizenmaier K., Krönke M. (1989). Regulation of tumor necrosis factor gene expression in colorectal adenocarcinoma: in vivo analysis by in situ hybridization.. Proc Natl Acad Sci U S A.

[PDF_00667] Davies B., Edwards S. W. (1992). Interactions between human monocytes and tumour cells. Monocytes can either enhance or inhibit the growth and survival of K562 cells.. Br J Cancer.

[PDF_00670] Ding A. H., Nathan C. F., Stuehr D. J. (1988). Release of reactive nitrogen intermediates and reactive oxygen intermediates from mouse peritoneal macrophages. Comparison of activating cytokines and evidence for independent production.. J Immunol.

[PDF_00674] Ernst M., Lange A., Flad H. D., Havel A., Ennen J., Ulmer A. J. (1984). Dissociation of responses measured by natural cytotoxicity and chemiluminescence.. Eur J Immunol.

[PDF_00677] Galligioni E., Quaia M., Spada A., Favaro D., Santarosa M., Talamini R., Monfardini S. (1993). Activation of cytolytic activity in peripheral blood monocytes of renal cancer patients against non-cultured autologous tumor cells.. Int J Cancer.

[PDF_00681] Hara N., Ichinose Y., Asoh H., Yano T., Kawasaki M., Ohta M. (1992). Superoxide anion-generating activity of polymorphonuclear leukocytes and monocytes in patients with lung cancer.. Cancer.

[PDF_00684] Hasday J. D., Shah E. M., Lieberman A. P. (1990). Macrophage tumor necrosis factor-alpha release is induced by contact with some tumors.. J Immunol.

[PDF_00686] Hruby Z., Beck K. F. (1997). Cytotoxic effect of autocrine and macrophage-derived nitric oxide on cultured rat mesangial cells.. Clin Exp Immunol.

[PDF_00689] Ioannidis I., de Groot H. (1993). Cytotoxicity of nitric oxide in Fu5 rat hepatoma cells: evidence for co-operative action with hydrogen peroxide.. Biochem J.

[PDF_00694] Jänicke R., Männel D. N. (1990). Distinct tumor cell membrane constituents activate human monocytes for tumor necrosis factor synthesis.. J Immunol.

[PDF_00692] Jättelä M., Wissing D. (1993). Heat-shock proteins protect cells from monocyte cytotoxicity: possible mechanism of self-protection.. J Exp Med.

[PDF_00697] King C. C., Jefferson M. M., Thomas E. L. (1997). Secretion and inactivation of myeloperoxidase by isolated neutrophils.. J Leukoc Biol.

[PDF_00699] Klassen D. K., Sagone A. L. (1980). Evidence for both oxygen and non-oxygen dependent mechanisms of antibody sensitized target cell lysis by human monocytes.. Blood.

[PDF_00702] Lanza F., Fietta A., Spisani S., Castoldi G. L., Traniello S. (1987). Does a relationship exist between neutrophil myeloperoxidase deficiency and the occurrence of neoplasms?. J Clin Lab Immunol.

[PDF_00705] Leek R. D., Lewis C. E., Whitehouse R., Greenall M., Clarke J., Harris A. L. (1996). Association of macrophage infiltration with angiogenesis and prognosis in invasive breast carcinoma.. Cancer Res.

[PDF_00708] Mantovani A., Bottazzi B., Colotta F., Sozzani S., Ruco L. (1992). The origin and function of tumor-associated macrophages.. Immunol Today.

[PDF_00710] Martin J. H., Edwards S. W. (1993). Changes in mechanisms of monocyte/macrophage-mediated cytotoxicity during culture. Reactive oxygen intermediates are involved in monocyte-mediated cytotoxicity, whereas reactive nitrogen intermediates are employed by macrophages in tumor cell killing.. J Immunol.

[PDF_00715] Mavier P., Edgington T. S. (1984). Human monocyte-mediated tumor cytotoxicity. I. Demonstration of an oxygen-dependent myeloperoxidase-independent mechanism.. J Immunol.

[PDF_00718] McLachlan J. A., Serkin C. D., Morrey K. M., Bakouche O. (1995). Antitumoral properties of aged human monocytes.. J Immunol.

[PDF_00720] McNally J. A., Bell A. L. (1996). Myeloperoxidase-based chemiluminescence of polymorphonuclear leukocytes and monocytes.. J Biolumin Chemilumin.

[PDF_00723] Nakabo Y., Pabst M. J. (1997). C2-ceramide and C6-ceramide inhibited priming for enhanced release of superoxide in monocytes, but had no effect on the killing of leukaemic cells by monocytes.. Immunology.

[PDF_00729] Nathan C. F., Silverstein S. C., Brukner L. H., Cohn Z. A. (1979). Extracellular cytolysis by activated macrophages and granulocytes. II. Hydrogen peroxide as a mediator of cytotoxicity.. J Exp Med.

[PDF_00726] Nathan C., Cohn Z. (1980). Role of oxygen-dependent mechanisms in antibody-induced lysis of tumor cells by activated macrophages.. J Exp Med.

[PDF_00732] Palomba L., Sestili P., Cattabeni F., Azzi A., Cantoni O. (1996). Prevention of necrosis and activation of apoptosis in oxidatively injured human myeloid leukemia U937 cells.. FEBS Lett.

[PDF_00735] Pazdrak K., Justement L., Alam R. (1995). Mechanism of inhibition of eosinophil activation by transforming growth factor-beta. Inhibition of Lyn, MAP, Jak2 kinases and STAT1 nuclear factor.. J Immunol.

[PDF_00737] Pericle F., Sconocchia G., Titus J. A., Segal D. M. (1996). CD44 is a cytotoxic triggering molecule on human polymorphonuclear cells.. J Immunol.

[PDF_00740] Pick E., Mizel D. (1981). Rapid microassays for the measurement of superoxide and hydrogen peroxide production by macrophages in culture using an automatic enzyme immunoassay reader.. J Immunol Methods.

[PDF_00743] Rothe G., Gabriel H., Kovacs E., Klucken J., Stöhr J., Kindermann W., Schmitz G. (1996). Peripheral blood mononuclear phagocyte subpopulations as cellular markers in hypercholesterolemia.. Arterioscler Thromb Vasc Biol.

[PDF_00747] Siedlar M., Stachura J., Szczepanik A., Popiela T., Mattei M., Vendetti S., Colizzi V., Zembala M. (1995). Characterization of human pancreatic adenocarcinoma cell line with high metastatic potential in SCID mice.. Invasion Metastasis.

[PDF_00753] Sun Y., Oberley L. W., Elwell J. H., Sierra-Rivera E. (1989). Antioxidant enzyme activities in normal and transformed mouse liver cells.. Int J Cancer.

[PDF_00758] Trulson A., Nilsson S., Brekkan E., Venge P. (1994). Patients with renal cancer have a larger proportion of high-density blood monocytes with increased lucigenin-enhanced chemiluminescence.. Inflammation.

[PDF_00756] Trulson A., Nilsson S., Venge P. (1989). Lucigenin-enhanced chemiluminescence in blood is increased in cancer.. Am J Clin Pathol.

[PDF_00761] Utsugi T., Schroit A. J., Connor J., Bucana C. D., Fidler I. J. (1991). Elevated expression of phosphatidylserine in the outer membrane leaflet of human tumor cells and recognition by activated human blood monocytes.. Cancer Res.

[PDF_00771] Zembala M., Czupryna A., Wieckiewicz J., Jasinski M., Pryjma J., Ruggiero I., Siedlar M., Popiela T. (1993). Tumour-cell-induced production of tumour necrosis factor by monocytes of gastric cancer patients receiving BCG immunotherapy.. Cancer Immunol Immunother.

[PDF_00776] Zembala M., Siedlar M., Marcinkiewicz J., Pryjma J. (1994). Human monocytes are stimulated for nitric oxide release in vitro by some tumor cells but not by cytokines and lipopolysaccharide.. Eur J Immunol.

[PDF_00779] Zembala M., Siedlar M., Ruggiero I., Wieckiewicz J., Mytar B., Mattei M., Colizzi V. (1994). The MHC class-II and CD44 molecules are involved in the induction of tumour necrosis factor (TNF) gene expression by human monocytes stimulated with tumour cells.. Int J Cancer.

[PDF_00764] van Muijen G. N., Danen E. H., Veerkamp J. H., Ruiter D. J., Lesley J., van den Heuvel L. P. (1995). Glycoconjugate profile and CD44 expression in human melanoma cell lines with different metastatic capacity.. Int J Cancer.

